# Intra-articular platelet-rich plasma vs corticosteroids in the treatment of moderate knee osteoarthritis: a single-center prospective randomized controlled study with a 1-year follow up

**DOI:** 10.1186/s13018-020-01753-z

**Published:** 2020-07-10

**Authors:** Andrejs Elksniņš-Finogejevs, Luis Vidal, Andrejs Peredistijs

**Affiliations:** 1 Faculty of Continuing Education, Rīga Stradiņš University, Riga, Latvia; 2 “ORTO klinika” Ltd., Riga, Latvia; 3 Laboratorios Fidia Farmacéutica S.L.U, Madrid, Spain

**Keywords:** Osteoarthritis, Knee, Platelet-rich plasma, Corticosteroid

## Abstract

**Background:**

Osteoarthritis is the most prevalent type of arthritis, which significantly impacts the patient’s mobility and quality of life. Pharmacological treatments for osteoarthritis, such as corticosteroids, produce an immediate reduction of the patient’s pain as well as an improvement in the patient’s mobility and quality of life, but with a limited long-term efficacy. In this context, platelet-rich plasma (PRP) infiltrations represent a therapeutic tool due to its trophic properties and its ability to control inflammatory processes, especially in musculoskeletal applications. The aim of this study is to evaluate and compare the clinical benefits of PRP when injected intra-articularly vs a commonly used corticosteroid (CS, triamcinolone acetonide, Kenalog®) in patients affected by mild to moderate symptomatic knee osteoarthritis.

**Methods:**

Forty patients affected by symptomatic radiologically confirmed knee osteoarthritis (Kellgren-Lawrence grades II–III) were enrolled in this randomized study. Patients randomized in the PRP group (*n* = 20) received an intra-articular injection of PRP (8 mL) while patients randomized in the CS group (*n* = 20) received an intra-articular injection of triamcinolone acetonide (1 mL of 40 mg/mL) plus lidocaine (5 mL of 2%). The pain and function of the target knee were evaluated by the VAS, IKDC, and KSS scales at the baseline (V1), 1 week (V2), 5 weeks (V3), 15 weeks (V4), 30 weeks (V5), and 1 year (V6) after treatment.

**Results:**

No serious adverse effects were observed during the follow-up period. A mild synovitis was registered in 15 patients (75%) in the PRP group within the first week after treatment which resolved spontaneously. Both treatments were effective in relieving pain and improving the knee function in the very short-term follow-up visit (1 week). A high improvement of the subjective scores was observed for both groups up to 5 weeks, with no significative differences between the groups for the VAS, IKDC, or KSS. After 15 weeks of follow-up, the PRP group showed significative improvements in all scores when compared to the CS group. Overall, the patients who received PRP treatment had better outcomes in a longer follow-up visit (up to 1 year) than those who received CS.

**Conclusions:**

A single PRP or CS intra-articular injection is safe and improves the short-term scores of pain and the knee function in patients affected by mild to moderate symptomatic knee OA (with no significant differences between the groups). PRP demonstrated a statistically significant improvement over CS in a 1-year follow-up. This study was registered at ISRCTN with the ID ISRCTN46024618.

## Background

Osteoarthritis (OA) is a slowly progressive chronic disease characterized by pain, loss of function, and deformity of the affected joints. In the past, OA was considered a normal sign of aging and it was described as a degenerative disorder that mainly causes cartilage loss [1]. However, more recent studies have shown that OA occurs and evolves due to the interaction of multiple risk factors affecting the whole joint including the cartilage, subchondral bone, synovium, ligaments, and menisci [2]. Currently, no disease-modifying treatment has been approved, which makes joint replacement the only viable solution for these patients. Non-pharmacological treatments include patient education and self-management, exercises, weight reduction, walking supports (crutches), bracing, shoe and insole modification, local cooling/heating, acupuncture, and electromagnetic therapy [3, 4]. Pharmacological treatments include topical and oral NSAID [5], intra-articular (IA) injections of corticosteroids, visco-supplements, and blood-derived products, including platelet-rich-plasma (PRP) (highly recommended when the use of oral analgesics or anti-inflammatories fails to release disease symptoms) [6]. The intra-articular (IA) infiltration of corticosteroids provides a short-term reduction in OA pain, and it is considered as an adjunct to core treatment for the relief of moderate to severe pain in people with OA [7]. This approach, however, has limited efficacy in delaying disease progression, as well as undesirable potential side effects when administered in high doses and frequency [8, 9]. In this context, PRP is proposed as a potential treatment, capable of improving the clinical condition of patients with osteoarthritis [10–15]. A limited number of publications in PRP, in which PRP has been compared to corticosteroid for the treatment of early knee OA, are available in the literature [16–18]. To address these concerns (what concerns, OA or the side effects, not clear), this study was designed to compare the efficacy of a single intra-articular dose of PRP compared to corticosteroids for the treatment of moderate knee OA. The objective of this study is to evaluate the clinical benefits of PRP when injected into the intra-articular space compared to a corticosteroid (triamcinolone acetonide, Kenalog®), with is a recognized pharmacological treatment in patients with mild to moderate symptomatic knee OA. We hypothesized that intra-articular injection of PRP reduces pain in a very short term (1-week follow-up), similar to triamcinolone acetonide [19], and it leads to an equal or more effective analgesic outcome plus better functional recovery at 1 year follow-up.

## Methods

### Participants

Demographic variables such as age, sex, body mass index (BMI), and the degree of radiological involvement were collected. A total of 40 patients (32 females and 8 males) with symptomatic, radiologically confirmed, knee OA (Kellgren-Lawrence grades II–III) were enrolled in a prospective, randomized, controlled study in the period from April 2016 to May 2017. The patient’s inclusion criteria were over 55 years of age, chronic pain history, swelling, and/or reduced range of motion in the knee joint. Clinical and radiological confirmation of knee’s OA (Kellgren-Lawrence grades II–III) were verified by X-ray images in anteroposterior and lateral projections. The patient’s exclusion criteria were post-traumatic knee osteoarthritis, pregnancy, breastfeeding, oncological diseases, endocrine diseases (gout, diabetes), autoimmune diseases (rheumatoid arthritis) acute/chronic infectious disease, blood clotting disorders (thrombocytopenia, coagulopathy), previous interventions on the knee joint (i.e., punctures, blockades, arthroscopy), and previous consistent hormonal therapy or non-steroidal anti-inflammatory drugs (NSAIDs) treatment (within 10 days prior to the intervention).

### Study design, randomization, and intervention

This was a single-center prospective randomized controlled study. Potentially eligible patients with knee pain were pre-screened. Patients, who signed an informed consent and met the inclusion criteria, were considered eligible and assigned in a 1:1 ratio into two groups. The patients were randomized using a computer-generated randomized list. Patients assigned to group one (platelet-rich plasma (PRP)) received one intra-articular injection of autologous PRP. Patients assigned to group two (corticosteroid (CS)) received one intra-articular injection of corticosteroid. The variation from the pain baseline, measured by the VAS score at 1 year (V1), was considered the primary outcome. The VAS pain score was self-completed by the patient. The patient was asked to place a line, perpendicular to the VAS line from the questionnaire, at the point that showed their pain intensity score in their last 7 days of daily activities (walking, working, home activities, house cleaning, and others). Secondary outcomes were the variations in VAS scores, the International Knee Documentation Committee (IKDC 2000 form) score, and the Knee Society Score (KSS) [20] at any time point of the study. All procedures performed in the studies involving human participants were approved by the Latvian local ethics committee and the national health regulatory authority of Latvia. All procedures performed in studies involving human participants were in accordance with the ethical standards of Ethics Committee for Medical and Biomedical Research, Rigas Stradins University (RSU) Ethics Committee, Ref E-9(2), and Riga Eastern Clinical University Hospital Support Foundation. This study was registered at ISRCTN (International Standard Randomized Controlled Trial Number) with the ID ISRCTN46024618, and it was carried out in accordance with the 1964 Declaration of Helsinki. All the patients were informed, before participating in the CT, of the risks of both treatments (including the beneficial and potential adverse effects). Informed consent was obtained from all individual participants included in the study.

### PRP preparation method

PRP was prepared using the Hy-Tissue PRP® system, a CE-marked medical device (Fidia, Abano Terme, Italy). To prepare PRP, 18 mL of peripheral blood was collected and 2 mL of 3.8% sodium citrate was added. In order to separate blood components according to their different specific densities, 20 mL of citrated blood was centrifuged at 1800 rpm for 8 min using a Duografter® II centrifuge (Fidia, Abano Terme, Italy). From this resulting plasmatic fraction, 8 mL of pure PRP solution was obtained and used for the intra-articular PRP injection.

### Infiltration

Patients in the first group received 8 mL of an intra-articular infiltration of PRP, and patients in the second group received an intra-articular infiltration of 1 mL of 40 mg/mL triamcinolone acetonide (Kenalog®) and 5 mL of 2% lidocaine mixed in a single syringe. Arthrocentesis was permitted in both study groups. All the baseline and follow-up visits were performed by an evaluator who was blinded to the treatment throughout the study. The intra-articular knee injection was performed under sterile conditions, without any local or general anesthesia, with a 20-G × 2.75 70 mm needle using an anterolateral approach. Echographic control (Philips Affinity 70) allowed the correct needle positioning by direct visualization of the PRP/CS liquid injected. After this manipulation, an aseptic cool bandage was applied, for 15 min, for local compression. Non-steroidal anti-inflammatory drugs were prohibited for 10 days following the injection. During the follow-up period, patients carried on their ordinary lives without any specific treatments or restrictions.

### Statistical analysis

The sample size calculation used the hypothesis of superiority. The pain was assessed on a visual analog scale (VAS; range 0–10 points) 12 months after the procedure. An average score of 7.3 was assumed in the control group with a standard deviation of 1.6. This meant that detecting a reduction of 1.5 points in the treatment group vs the control group with a power of 80% and 2-sided significance level of 0.05 would require the inclusion of a total of 36 patients. Considering a possible dropout rate of 10%, 40 patients in total were required (20 patients per group). A difference in the VAS of 1.5 points for the average score and a standard deviation between the 2 groups was fixed (based on published results) [16]. The primary and secondary variables were analyzed using the intention-to-treat principle. Categorical variables were described by percentages and frequencies while continuous variables were described by means, standard deviations, and the 95% confidence interval of the mean. Parametric tests (unpaired *t* test) were used for normal distributions and the Mann-Whitney *U* test for non-parametric distributions. Data symmetry was analyzed using a D’Agostino and Pearson normality test. Categorical variables were compared using chi-square tests. For all tests, *p* < 0.05 was considered statistically significant. Patient randomization was performed using the “Randomizer for Clinical Trial” software. All statistical analyses were performed using GraphPad Prism version 7.00 for Windows (GraphPad Software, La Jolla, CA, USA).

## Results

**Fig. 1:**
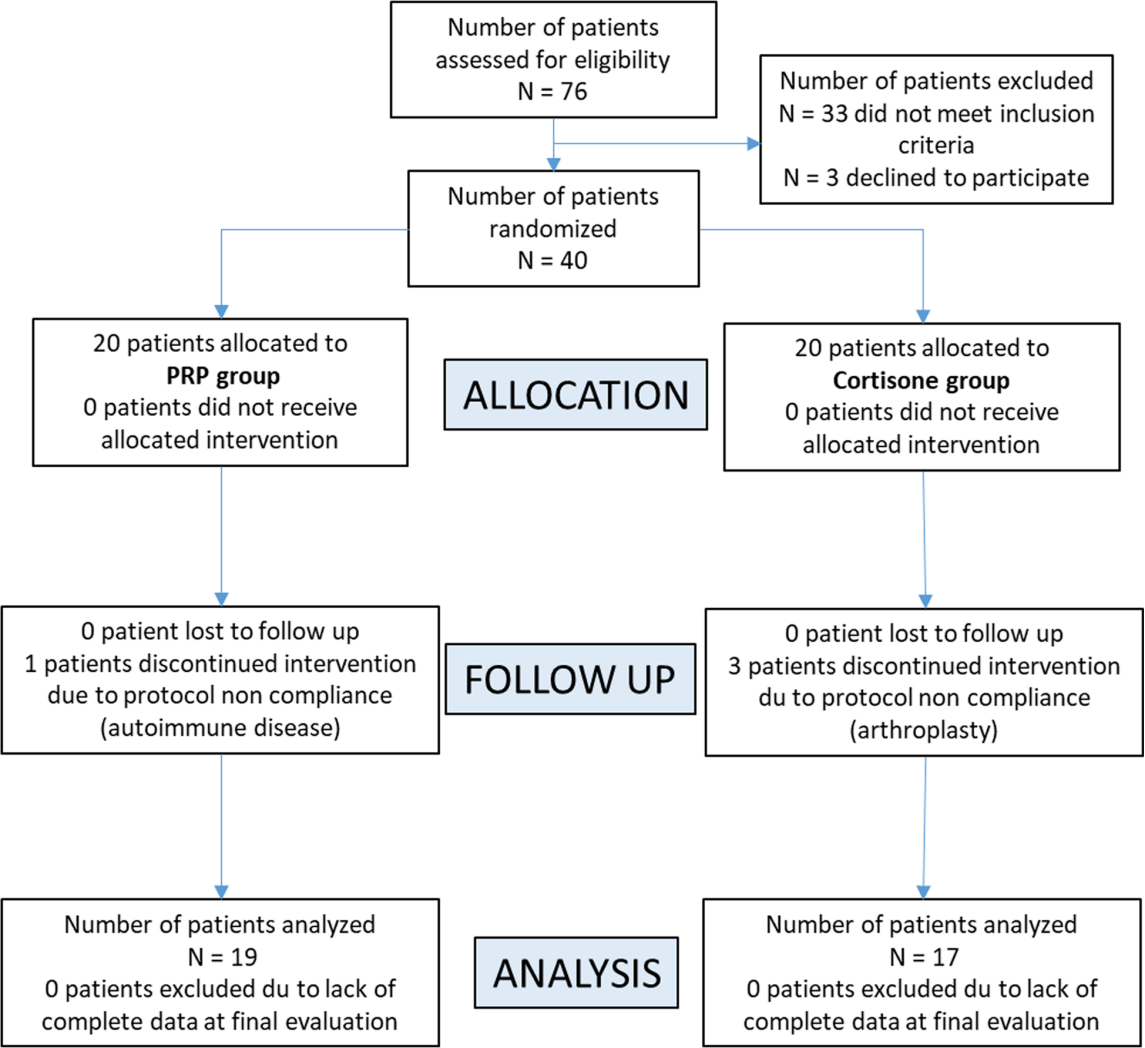
CONSORT (Consolidated Standards of Reporting Trials) flow diagram for the present study

A total of 76 patients were screened from April 2016 to May 2018. Of these screened patients, 33 patients did not meet inclusion criteria and 3 declined to participate (Fig. 1). After this, 40 patients were included in either group (per randomization). The mean age of the intention-to-treat patients was 66.5 ± 8.6 and 70.1 ± 9.1 for the PRP and CS group, respectively. Participants were 84% men and 16% women for the PRP group and 71% and 29% for the CS group, respectively. There were no significant differences between the 2 groups across all the baseline parameters (Table 1): age, sex, and K-L grade for OA (except for the basal IKDC index (*p* = 0.038)). According to the intention-to-treat population, 19 patients were included in the per-protocol evaluation for the PRP group and 17 in the CS group. A total of 4 patients (10%) that were randomized (one from the PRP group and three from the CS group) were not included in the analysis. One randomized patient into the PRP group discontinued the evaluation due to the presentation of an autoimmune disorder after 6 months. This patient presented red spots on the face that was later diagnosed with lupus erythematosus. Three patients that were treated with CS were unable to continue the trial due to an arthroplasty at 6, 7, and 9 months.

**Table 1:** Baseline characteristics of intent-to-treat patients included in the clinical trial

	PRP group (*N* = 20)	Corticosteroid group (*n* = 20)	*p* value
Gender, M:F, *n*	17:3	15:5	ns
Age, years, mean/SD	66.4 ± 8.4	70.2 ± 9.2	ns
BMI, mean/SD	28.6 ± 5.0	30.5 ± 5.8	ns
K-L degree (II/III), *n*	5:15	6:14	ns
Knee (right/left), *n* (%)	14/6 (70%/30%)	12/8 (60%/40%)	ns
VAS baseline, mean/SD	6.1 ± 1.2	6.0 ± 1.4	ns
KSS baseline, mean/SD	58.3 ± 7.2	54.0 ± 8.2	ns
IKDC baseline, mean/SD	36.6 ± 10.4	30.0 ± 8.8	0.0377

Data are provided as mean ± SD (range), unless indicated otherwise

*BMI* body mass index, *K-L*, Kellgren-Lawrence classification radiographically confirmed, *VAS* visual analog scale, *KSS* Knee Society Score, *IKDC* International Knee Documentation Committee, *ns* not significant

### Clinical results

Both PRP and CS single injections were effective in reducing pain, and they improved the knee function after the first week of treatment. VAS score changes at 1 year (primary clinical outcome) showed a higher mean change from baseline in the PRP group than the CS group (PRP − 3.1 ± 2.0, − 52%; CS − 0.8 ± 1.8, − 14%). This difference was significant between groups (*p* = 0.0002). The most surprising effect observed was that PRP induced pain relief just as fast as CS. In fact, a significant reduction of pain from baseline for both groups was found 1 week after treatment (mean VAS change—PRP − 2.8 ± 2.3, − 47%; CS − 3.4 ± 1.2%, − 58%; *p* < 0.0001). Similarly, significant function improvements from baseline were obtained in the first week for both treatment groups (mean IKDC change—PRP 22.1 ± 16.9, 60%; CS 35.4 ± 10.0, 117%—and mean KSS change—PRP 22.7 ± 12.3, 39%; CS 29.4 ± 12.8, 55%). Interestingly, the pain reduction and the knee functional improvement were not significant between both groups in the very short-term follow-up visit (up to 5 weeks; Table 2). The highest change in the VAS score from the baseline was at 3 months for the PRP group (mean − 4.6 ± 1.6; − 77%) and at 1 month in the CS group (− 3.4 ± 1.2; − 58%).

**Table 2:** Primary outcome and secondary outcomes in per-protocol population during the follow-up of the study

		PRP group (*n* = 19)		CS group (*n* = 17)			
	Weeks	Mean ± SD	CI (95%)	Mean ± SD	CI (95%)	*p* value (intergoup)	
**A**							
**VAS**							
V1	0	6.1 ± 1.3	5.4–6.6	6.0 ± 1.5	5.2–6.8	0.9855	ns
V2	1	3.2 ± 2.1	2.2–4.2	2.5 ± 1.7	1.6–3.3	0.3675	ns
V3	5	2.3 ± 1.8	1.4–3.2	2.5 ± 1.5	1.7–3.2	0.6525	ns
V4	15	1.4 ± 1.2	0.8–2.0	3.6 ± 2.1	2.5–4.7	0.001	***
V5	30	1.6 ± 1.9	0.7–2.6	4.0 ± 1.6	3.2–4.8	< 0.0001	****
V6	58	2.9 ± 1.5	2.2–3.6	5.1 ± 1.9	4.1–6.0	0.0008	***
**B**							
**IKDC**							
V1	0	36.3 ± 10.7	31.2–41.4	28.9 ± 8.3	24.6–33.1	0.0570	ns
V2	1	61.2 ± 14.4	54.6–67.4	65.9 ± 13.4	59.0–72.8	0.1925	ns
V3	5	68.8 ± 14.8	61.7–75.9	64.1 ± 17.4	55.2–73.0	0.3747	ns
V4	15	78.7 ± 11.4	73.3–84.2	58.2 ± 15.9	50.1–66.4	0.0004	***
V5	30	77.5 ± 14.2	70.6–84.3	56.3 ± 17.4	47.4–65.3	0.0008	***
V6	58	62.0 ± 15.6	54.5–69.6	39.8 ± 16.3	32.8–46.8	0.0002	***
**C**							
**KSS**							
V1	0	57.8 ± 7.1	54.4–62.2	53.2 ± 8.4	48.8–57.5	0.0998	ns
V2	1	81.3 ± 12.6	75.2–87.4	83.2 ± 9.9	78.1–88.4	0.7598	ns
V3	5	85.7 ± 10.5	80.6–90.7	80.9 ± 11.0	75.3–86.6	0.1925	ns
V4	15	88.8 ± 9.4	84.2–93.3	73.2 ± 13.4	66.2–80.0	0.0013	**
V5	30	86.8 ± 11.9	81.1–92.5	71.2 ± 13.7	64.2–78.3	0.0008	***
V6	58	77.3 ± 12	71.6–83.1	60.3 ± 13.7	53.3–67.3	0.0004	***

Data are provided as mean ± SD (range)

*p* value of Mann-Whitney test

*ns* not significant, *VAS* visual analog scale, *KSS* Knee Society Score, *IKDC* International Knee Documentation Committee, *CI* confidence interval

** Statistically significant difference (*p* ≤ 0.01)

*** Statistically significant difference (*p* ≤ 0.001)

**** Statistically significant difference (*p* ≤ 0.0001)

**Fig. 2:**
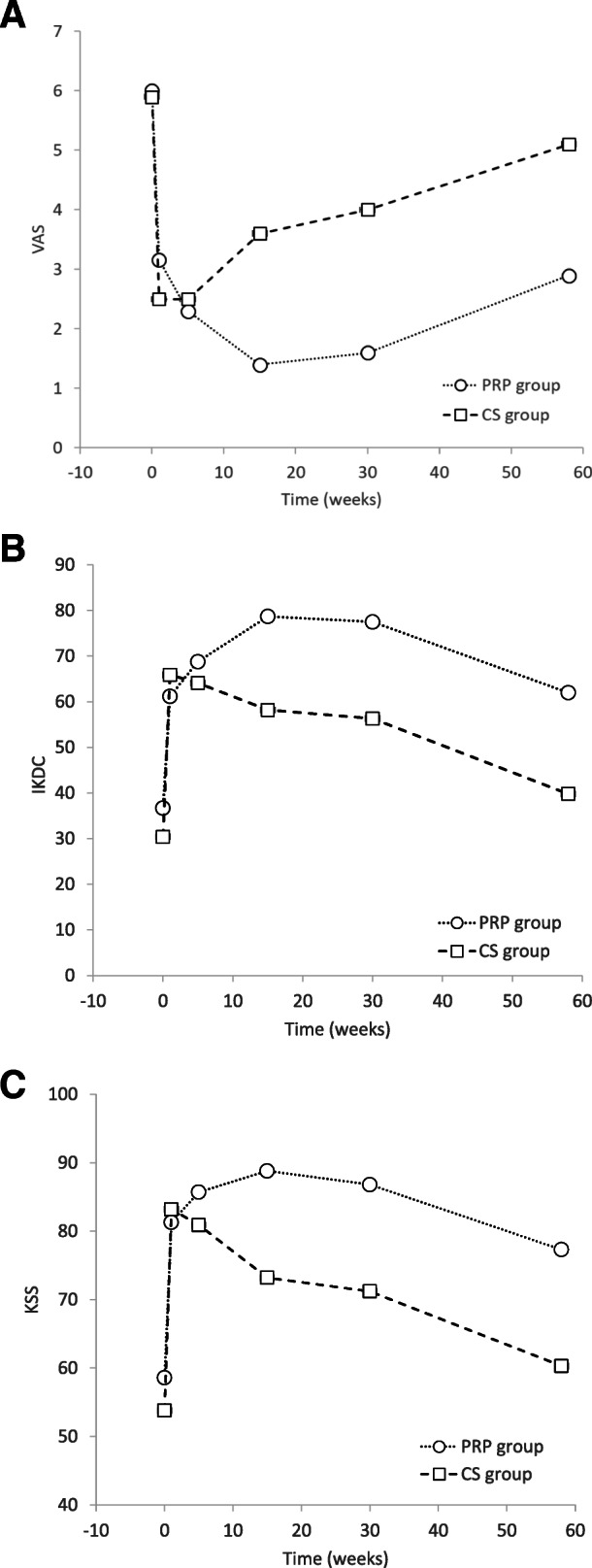
Evaluation of the subjective state according to VAS (**a**), IKDC (**b**), and KSS (**c**) over time. Values are the average of each score at any one time. VAS, visual analog scale; KSS, Knee Society Score; IKDC, International Knee Documentation Committee

The pharmacological effect of CS seemed to disappear 15 weeks after receiving treatment as all scores tended to worsen after this period. For instance, pain in the CS group improved rapidly but, in general, worsened after 15 weeks of treatment, and the pain steadily increased in each follow-up visit. At the same time, the PRP group resulted in a sustained improvement in pain relief up to 30 weeks, showing a small increase in pain in the 1-year evaluation follow-up (Fig. 2a). For all other outcome scores, there were significant differences between pre-treatment and post-treatment results at any time, evaluated up to 58 weeks of the follow-up (*p* < 0.05), except for the VAS (*p* = 0.1537) and KSS (*p* = 0.1719) indexes for the CS group at 58 weeks (due to worsening of the pain conditions of the patients).

Knee function improvement was observed in both groups up to 5–15 weeks with no significant differences between groups (*p* > 0.05) (Table 2). At V4 (15 weeks), the PRP group presented a better significant improvement in the IKDC and KSS scores compared to the CS group, which decreased in effectiveness up to 1 year (Fig. 2b, c). Maximum functional improvement and better patient expectation, satisfaction, and activity levels were observed after 15 weeks for the PRP group (mean change from baseline of 41.1 ± 13.6, 112% and 30.2 ± 11.7, 51% for IKDC) and after 5 weeks for the CS group (mean change from baseline of 33.7 ± 13.5, 111% and 29.4 ± 12.8, 55% for KSS).

### Safety

No serious adverse events (SAE) occurred. No adverse events were registered in the CS group. Mild synovitis was registered by 15 patients (75%) in the PRP group at the first week after treatment (diagnosed by ultrasound and clinical evaluation: patellar tap test, brush test, fluid displacement, and wave test) that resolved spontaneously. No synovitis was reported from the patients of the CS group.

## Discussion

This single-center prospective randomized controlled study showed that a single intra-articular injection of PRP was more efficient than CS for treating moderate OA (Kellgren-Lawrence grades II–III) compared to triamcinolone acetonide. The effectiveness of PRP has been questioned by some authors because the evidence of its efficacy has been highly variable depending on the specific indication [21–24]. Other studies have shown that PRP has been effective for knee OA when compared to placebo, ozone, or HA in several high-quality, randomized, controlled trials [25–29]. Some of these studies suggested that intra-articular infiltrations of PRP provide quantifiable benefits for pain relief and functional improvement within a limited time period (up to 1 year) [25, 28, 29]. For instance, Filardo et al. [30] performed three consecutive intra-articular infiltrations of PRP in a group of 91 patients with chronic degenerative knee conditions with improvement in the IKDC and EQ-VAS indexes up to 1 year. However, this condition deteriorated 24 months after 1 year of clinical improvements (especially for younger patients with a low to mild degree of cartilage degeneration). Similar results were reported by Fukawa et at [31]., who performed three consecutive injections of PRP with a VAS reduction up to 1 year with a deterioration of the improvement after 24 months. In this study, a single intra-articular injection of PRP resulted in significant pain relief for up to 12 months, with a maximum pain decrease after 3 months. This difference suggested that the number of PRP injections could be critical for the maintenance of the beneficial effect. In contrast to this, Patel et al. [32] did not find significant differences in WOMAC scores between single and double PRP injections for early OA at 6 weeks and 3 and 6 months.

As far as we know, only a few trials have been published that compare clinical outcomes after PRP and corticosteroid (CS) injections for treating mild OA [16–18]. In general, those studies concluded that a single dose of PRP is comparable to 1 dose of CS (none of them analyzed the very short-term effects—1 week). Most of the studies of PRP commonly perform the first assessment of patients 1 month after treatment, making impossible the determination of the evolution in the first few days [33]. Based on this remark and based on our personal experience that we have from our routine care activity, we evaluated the clinical outcome of the patients in the very short term (1 week). From our knowledge, this is the first PRP clinical study for early-stage knee OA that incorporated outcome assessment by means of this approach. We found that the analgesic effect of PRP in the very short term was comparable to CS, and the same was noticed for the knee function (Table 2; 1 to 5 weeks; ns). The rapid reduction in pain observed upon treatment with PRP might be attributed to a combined effect, mainly due to the induction of endogenous cell endocannabinoids [34] together with the anti-inflammatory activity effect of PRP on chondrocytes [35, 36]. The variation in pain and knee function in the PRP group contributed to the sustained duration of the overall beneficial effects for up to 30 weeks, whereas in the CS group, a tendency to worsen after 5 weeks was registered. A recent meta-analysis reported the limitation of the beneficial effect of the CS 15 weeks after the end of treatment [37]. We hypothesized that the improvement of both parameters (pain and function) were mainly due to control of the inflammation of the knee rather than the trophic effect of PRP on cartilage. This rationale is due to the fact that it has not yet been demonstrated that the improvement of knee function after PRP treatment correlates with a volume increase of the articular cartilage [38–40], even when has been demonstrated that in vitro, TGF-β1/3 can induce chondrogenesis of mesenchymal stem cells [41]. However, there is evidence that suggests that PRP has other effects on the joints other than the anti-inflammatory and this may probably explain why the group that received PRP had better results than the CS group. Even though the mechanism of action on improving cartilage repair remains unclear, it has been reported in the literature that PRP can induce tissue maturation characterized by increased cell proliferation and tissue stiffness [42]. These cells, in turn, produce more superficial zone protein that functions as a boundary lubricant that helps reduce friction and wear [43, 44]. Moreover, it has been reported that PRP can enhance HA secretion from synovial fibroblasts in arthritic patients, producing a lubricating effect that could reduce the shear stress of the joint [45].

Administration of CS for treating OA has been controversial because these injections can reduce pain in the short term, but they may not be helpful in the treatment of the underlying arthritic lesion [46]. CS administrations have been reported to have deleterious effects on musculoskeletal tissues such as reduction of collagen synthesis, suppression of cell proliferation, induction of oxidative stress, and impact on cell viability [47–50]. This harmful effect could also influence the difference in long-term results between treatment groups in detriment of the CS group.

Intra-articular PRP infiltrations have been widely used for the treatment of knee OA with many beneficial results [28, 32, 51]. In this study, intra-articular PRP injections were well tolerated. The most common side effect being mild synovitis tended to resolve within the first week after treatment. Treatment with PRP injections can be considered safe since no severe adverse events or complications have been reported. We consider that the safety of PRP is mainly due to two factors: the administration of the PRP done through minimally invasive procedures and non-existent risk of transmission of infectious diseases (because it is an autologous procedure). In addition, in this study, we used pure PRP (the leukocyte layer fraction was not recovered per manufacturer’s instructions) to prevent possible inflammatory reactions [52, 53] in spite of the reported safety of using leucocyte-rich PRP [54]. Another relevant aspect of this study was that we plotted our results on a numerical scale (Fig. 2). Curiously, most of the studies represent the time variable on non-numerical plots which masked the real trends. This makes it difficult to appreciate the rate of the clinical improvement or worsening. For instance, outcome evolution graphs with ordinate axes (*X*) with equidistant values of 1, 3, and 6 months are frequently found in the literature [38, 55, 56].

### Limitations

The major limitation of the study was the absence of double-blinding. Blinding was not possible for the patients because the PRP preparation requires an additional blood withdrawal that would not be required for the CS group. Blinding was not possible for the clinicians who performed the infiltration because of the visible different aspects of the two compounds. Also, the number of patients included in this trial might have been underestimated. Although a sample size calculation was performed based on the assumption of an expected improvement of 1.5 points in VAS pain in the treatment group vs the control group at 1 year after treatment, and given that pain is a very subjective variable, the sample might need to be larger. Moreover, the loss of 4 patients due to causes not related to the treatment (1 for the PRP group and 3 for the CS group) could be relevant for the analysis of the clinical results. Other limitations were the absence of magnetic resonance imaging (MRI) data which is an important objective measurement to determine the potential effect of PRP on the cartilage tissue. Another limitation was that the baseline IKDC scores showed statistically significant differences between groups, maybe due to due to the low sample size and that could induce bias in the analysis of the results.

## Conclusion

This study shows that one intra-articular PRP injection is safe, it can reduce pain, and it can improve the knee function of patients with mild/moderate knee OA. The PRP intra-articular injection improves short-term scores of pain and knee function with no significant difference when compared to corticosteroids. However, PRP treatment resulted in a longer sustained effect than triamcinolone up to 12 months after administration. PRP can also produce a mild synovitis in the first week after administration without risk for the patient. It is recommended that a larger, randomized clinical trial should be performed to further assess the efficacy of PRP treatment in patients with mild/moderate OA.

## Data Availability

Data is available via the corresponding author under a reasonable request.

## Abbreviations

BMI: Body mass index
Cs: Corticosteroid
IA: Intra-articular (IA)
IKDC: International Knee Documentation Committee
ISRCTN: International Standard Randomized Controlled Trial Number
KSS: Knee Society Score
MRI: Magnetic resonance imaging
NSAIDs: Non-steroidal anti-inflammatory drugs
OA: Osteoarthritis
PRP: Platelet-rich plasma
SAE: Serious adverse events
TGF: Transforming growth factor
VAS: Visual analog scale
